# Distance Matters: Assessing the Influence of Spatial Separation on Reproductive Success of *Costus spiralis* (Costaceae) in a Vereda Palm Swamp

**DOI:** 10.3390/plants14213266

**Published:** 2025-10-26

**Authors:** Jessyca Santana Santos, Rafaela Cabral Marinho, Clesnan Mendes-Rodrigues, Monize Altomare, Paulo Eugênio Oliveira

**Affiliations:** 1 Instituto de Biologia, Universidade Federal de Uberlândia, Uberlândia 38405-320, Minas Gerais, Brazil; jessycasantanadossantos@gmail.com (J.S.S.); clesnan@ufu.br (C.M.-R.); monize.altomare@unesp.br (M.A.); 2 Instituto Acadêmico de Ciências da Saúde e Biológicas, Universidade Estadual de Goiás, Itumbiara 75536-100, Goiás, Brazil; 3 Enfermagem, Faculdade de Medicina, Universidade Federal de Uberlândia, Uberlândia 38405-320, Minas Gerais, Brazil; 4 Center for Research on Biodiversity Dynamics and Climate Change and Department of Biodiversity, Phenology Lab, Biosciences Institute, UNESP—São Paulo State University, Rio Claro 13506-900, São Paulo, Brazil

**Keywords:** pollen dispersal, reproductive success, *Costus spiralis*, vereda palm swamp, hummingbird pollination

## Abstract

Many plant species depend on pollen flow to maximize reproduction and maintain genetic variability. Pollinators mediate this process, but distance between individuals can influence its benefits. Proximity may cause inbreeding depression, while extreme distances can mix locally adapted genotypes, leading to outbreeding depression and reduced fitness. Vereda palm swamps, shaped by the water table, are important habitats in the Cerrado, but they face anthropogenic changes that can affect reproduction, pollinators, and genetic diversity. This study examined how pollen dispersal distance influences the reproductive success of spiral ginger (*Costus spiralis*, Costaceae), a vereda self-compatible rhizomatous herb pollinated by hummingbirds. Hand pollinations were carried out between plants ranging from 10 to 2000 m distant, and the resulting fruit-set and seed quality traits were evaluated. Fruit set did not vary significantly with distance, with no hint of either inbreeding or outbreeding depression. Nevertheless, seeds resulting from shorter pollination distance (10 m) were heavier, while germination rates were up to five-fold higher at greater pollination distances than at shorter pollination distances. These distinct seed traits are possibly related to main hummingbird pollinators of *C. spiralis*, which show distinct foraging strategies. The results suggest that *C. spiralis* is adapted to various modes of pollen dispersal, ensuring reproduction via either territorial or traplining hummingbirds.

## 1. Introduction

Plants have reproductive strategies that maximize success in dynamic environmental conditions [1] Such strategies modulate pollen flow mediated by pollinators, between neighboring or distant plants, between flowers within the same individual or even within a single flower [2,3,4]. Pollination influences the reproductive success and affects both the entire population’s genetic structure and its persistence in each area [5]. Besides flower structure and attractiveness, pollen flow can be influenced by the spatial distribution of individuals within a population [6,7,8]. The distance between individuals can significantly impact the probability and benefits of cross-pollination. When individuals are close, they tend to be genetically related, thereby increasing the chances of inbreeding [9]. On the other hand, mating between genetically distinct, more distant individuals can generate genetic variability. Nevertheless, long-distance pollen flow can reduce fitness since individuals may be genetically incongruent [10,11] These effects on plant reproductive fitness have been referred to as inbreeding depression and outbreeding depression [9,12] and may help in understanding plant survival ability and pollination requirements. At the population level, there are still relatively few studies on the consequences of spatial distribution for plant reproductive success [12,13,14,15].

Nevertheless, these consequences depend on other factors besides distance, such as pollinator behavior during pollen collection and deposition, and seed dispersal within the population [11,16,17,18,19]. Therefore, pollinator behavior and flight ability can affect gene flow. Pollinators with limited mobility can restrict gene flow within populations [20,21], while those with greater flight capacity can travel longer distances and are therefore more likely to increase genetic variability [21]. In this sense, plants pollinated by insects exhibit higher genetic differentiation at the population level when compared with other plants pollinated by hummingbirds, possibly due to long-distance pollen flow provided by these agile birds [21]. Even different types of visitors to the same plant species can vary considerably in behavior and distances traveled, consequently affecting pollen flow and quality [16,22].

Another factor that can also affect pollen flow is the density of flowers and available floral resources within populations and communities. For instance, hummingbird foraging behavior is influenced by flower and resource density, which changes flight distance and patch use [22,23,24,25]. Differences in density, ecological conditions and availability of resources along corridors or linear patches of forest or other plant formations may facilitate pollinator foraging and connect otherwise isolated plants in a matrix of distinct or disturbed plant formations [19,26,27]. Schulke and Waser (2001) [19] observed that the few *Delphinium nuttallianum* flowers, present in the meadows during their experiment, influenced the behavior of generalist pollinators, altering their foraging route to find isolated individuals. Thus, the distribution of floral resources and the spatial structure of vegetation itself can be challenging for species that rely on pollen flow as part of their reproductive strategies to persist in heterogeneous environments such as vereda palm swamps in Central Brazil.

Veredas palm swamps are hygrophilous plant formations occurring amid a much drier and seasonal savanna matrix. Veredas are an important part of the Cerrado domain, the Neotropical savanna region in Central Brazil, and are directly linked to water resources, due to the upwelling of the water tables [28]. In the matrix of drier savanna areas, the veredas are linear plant formations along shallow valleys defined by the amount of organic matter and water availability [29,30,31]. The topographic gradient and soil moisture zones create floodable microhabitats with floristic and structural peculiarities [29,32,33,34]. For plants adapted to these flooded habitats, pollen flow is possibly limited to the linear paths across the surrounding matrix of seasonal savannas. Vereda plants distributed along these habitats, thus, must provide floral resources to attract pollinators to their specific habitats. These floristic peculiarities, added to environmental factors, highlight the uniqueness of the veredas and reinforce their importance not only in maintaining plant diversity, but also as essential for shelter, food and breeding grounds for Cerrado fauna [35,36].

However, vereda areas have been subject to anthropogenic disturbances, which, combined with ongoing climate changes, can cause environmental degradation that results in alterations in the water regime and decreasing groundwater, leading to drought and irreversible changes in the vereda habitats [30,37,38,39]. These changes can also affect plant population structure, pollinators, pollen flow, fruit set and the reproductive strategies of vereda plants, which, in the long term, can interfere with the intrapopulation spatial genetic structure, genetic diversity and even lead to local extinction [40]. Therefore, a population approach to the reproductive success of vereda plants and its relationship with spatial distribution of individuals can bring relevant information on their structure and conservation.

The objective of our study was to test the effect of cross-pollinations between individuals at different spatial distances on the reproductive success of *Costus spiralis* (Jacq.) Roscoe (Costaceae), commonly known as spiral ginger, a rhizomatous herb typical of vereda habitats, considering the role of pollinators in mediating pollen flow. More specifically, our hand-pollination experiments sought to answer the following questions: (1) Does fruit and seed production vary with the spatial distance between pollen donors and recipients, potentially influenced by pollinator behavior? (2) Is there a particular distance at which reproductive success is maximized, possibly reflecting pollinator foraging patterns? (3) Does distance affect only fruit production, or does it also impact seed traits such as weight (mass) and germination, potentially through variations in pollinator effectiveness over different distances?

## 2. Results

**Figure 1:**
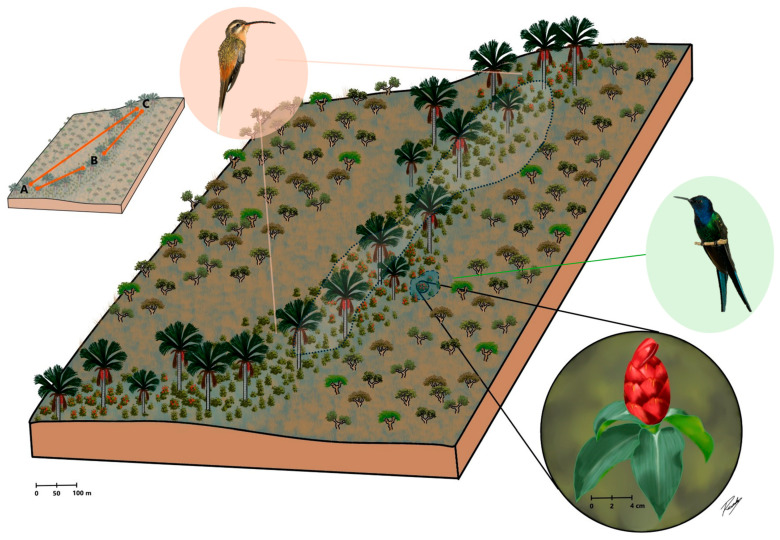
A representative diagram of the studied vereda palm swamp in Central Brazil surrounded by others Cerrado savannas physiognomies. *Costus spiralis* (red inflorescence in the lowest inset) is a rhizomatous herb which grows within waterlogged vereda microhabitats. Since genets were hard to distinguish in this rhizomatous species, we considered as a plant a cluster/clump of spouting ramets distant several meters from other neighbor clusters. The species is visited and pollinated by *Eupetomena macroura*, a territorial hummingbird (green right inset), and *Phaethornis pretrei*, a trapliner hummingbird (pink left inset). Hand cross-pollination treatments were carried out among plants at different distances (10 m and 60 m within A, B and C subpopulations), 800 m (between A and B subpopulations) and 2000 m (between A and C subpopulations). Pollinations between B and C subpopulations were not carried out. Hummingbird insets were adapted from Araújo et al. [41].

We investigated the effect of cross-pollination distance on the reproductive success of *C. spiralis* by conducting hand-pollination experiments in three different subpopulations in a vereda area (A, B and C, Figure 1; see Supplementary Materials Table S1 for specific cluster locations). Pollination treatments were as follows: 10 m (pollinations between nearby plants within a subpopulation), 60 m (pollination within a subpopulation but between plants at least 60 m but less than 100 m away), 800 m (pollination between subpopulation A and B plants) and 2000 m (pollinations between subpopulation A and C plants). A detailed description of procedures is provided in Section 4 and depicted in Figure 1.

**Figure 2:**
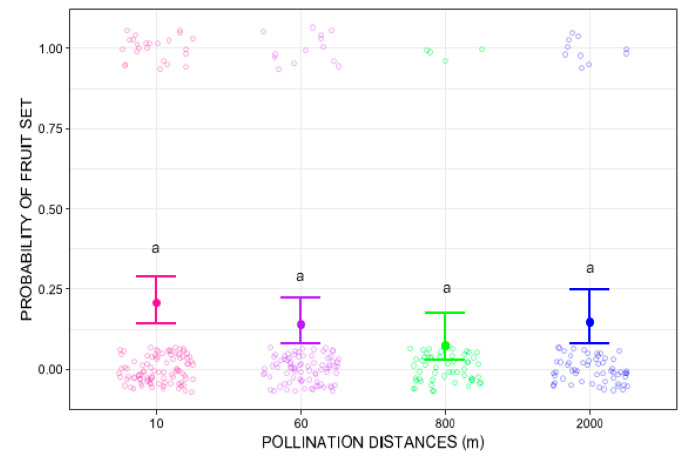
Probability of fruit set of *Costus spiralis* flowers after hand-pollination distance treatments (10 m, 60 m, 800 m, 2000 m) conducted within a vereda palm swamp in Uberlândia, Minas Gerais, Brazil. The solid dots and error bars represent the mean and 95% confidence intervals, respectively. Same letters above each distance treatment show no statistically significant differences (*p* < 0.05) after Tukey pairwise tests. The color circles jitter is created by the *ggplot2* package more for visual effect and does not represent proper data.

Pollination distance treatments did not significantly influence fruit-set (GLMM Wald chi-square = 5.2912, *p* = 0.1512). At 10 m, fruit set probability was 0.21 (95% CI: 0.14–0.29), with 33 fruits from 120 flowers. Slightly lower mean values were observed at 60 m (average fruit-set = 0.14, 95% CI: 0.08–0.22; 16 fruits in 120 flowers), 800 m (average fruit-set = 0.07, 95% CI: 0.03–0.18; 6 fruits in 120 flowers), and 2000 m (average fruit-set = 0.14, 95% CI: 0.08–0.25; 10 fruits in 120 flowers). Nevertheless, post hoc pairwise comparisons using Tukey’s test did not show any significant differences (Figure 2).

**Figure 3:**
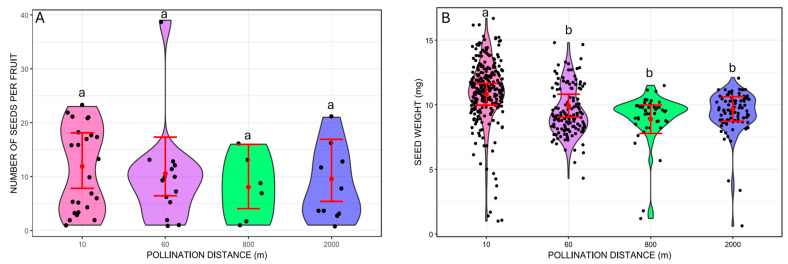
Traits of *Costus spiralis* seeds resulting from hand-pollination distance treatments (10 m, 60 m, 800 m, 2000 m) conducted within a vereda palm swamp in Uberlândia, Minas Gerais, Brazil. (**A**): Number of seeds per fruit from each distance treatment. (**B**): Weight of individual seeds (mg) formed from each distance treatment. Solid red dots and error bars represent the mean and 95% confidence intervals, respectively. Different letters above pollination distance treatments represent significant differences, *p* < 0.05, after Tukey pairwise tests.

We did not find significant differences in the number of seeds per fruit between the different pollination distances (Wald chi-square 1.48, *p* = 0.6865; Figure 3). On the other hand, seed mass varied from 1.2 mg to 16.7 mg and differed between pollination distance treatments (GLMM Wald chi-square 37.19, *p* < 0.001; Wald chi-square = 6.65, *p* = 0.0099 for the number of seeds per fruit included as a covariate). The treatment with the heaviest mean seed weight was at 10 m (mean = 10.82 mg, 95% CI: 9.98–11.66), which was significantly different from the other distances based on pairwise Tukey’s comparisons (*p* < 0.001 for all cases; Figure 3). The other distances did not differ from each other (*p* > 0.05 for all pairwise Tukey’s comparisons; Figure 3). The mean seed weights were 9.93 mg at 60 m (95% CI: 9.04–10.82), 8.89 mg at 800 m (95% CI: 7.79–9.99), and 9.67 mg at 2000 m (95% CI: 8.72–10.63).

**Figure 4:**
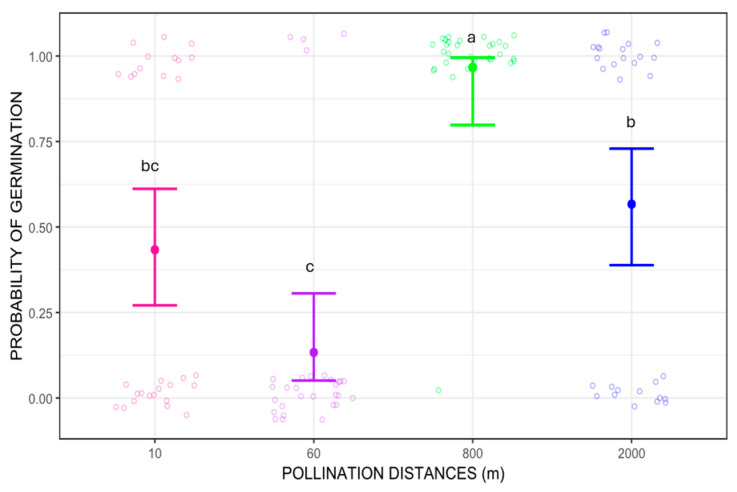
Probability of germination of *Costus spiralis* seeds resulting from different pollination distance treatments (10 m, 60 m, 800 m, 2000 m) in a vereda palm swamp in Uberlândia, Minas Gerais, Brazil. Solid dots and error bars represent the mean and 95% confidence interval, respectively. Different letters above each pollination distance treatment represent significant difference, *p* < 0.05, after Tukey pairwise tests. The jitter is created by the *ggplot2* package more for visual effect and does not represent proper data.

Regarding seed germination, the probability of germination differed between pollination distance treatments (Wald chi-square 51.618, *p* < 0.01). The distance with the highest germination success was 800 m, which showed significant differences when compared to 10, 60 m and 2000 m pollination treatments (*p* = 0.0043, *p* < 0.0001, and *p* = 0.0217 respectively for Tukey’s pairwise comparisons). When compared pairwise, seeds from 800 m pollination treatment germinated about five times more frequently than those from 60 m and three times more than 10 m and 2000 m (*p* = 0.00379 and *p* = 0.02002, respectively). Additionally, seeds from 2000 m pollination treatment had about twice the germination success than those from 60 m (*p* = 0.00526). The probability of germination was higher for seed from longer pollination distances, 800 m and 2000 m, ranging from 50% to 100%, while for shorter pollination distances, 10 m and 60 m, the probability of germination was below 50% (Figure 4). Despite the better germination success at longer distances, the overall reproductive output (RO), calculated as the probability of producing seedlings (probability of fruit-set multiplied by probability of germination), was estimated as RO = 0.12 for 10 m pollination distance, RO = 0.021 for 60 m, RO = 0.05 for 800 m, and RO = 0.04 for 2000 m.

No focal observations were conducted to quantify pollinator visits. However, throughout the experiment, we observed visits of *Eupetomena macroura* hummingbirds. They exhibited territorial behavior, defending clumps of *C. spiralis* plants from other potential visitors, systematically visiting flowers within a clump at regular intervals, and then perching on branches above the flowers in between visits. This behavior was observed several times during the mornings when the hand-pollination experiments were conducted. Visits from *Phaethornis petrei* were much rarer throughout the study period but this hermit hummingbird is commonly observed visiting *C. spiralis* in the area [42].

## 3. Discussion

*Costus spiralis* plants showed no fruit-set differences between pollination distance treatments. However, we did observe heavier seeds at shorter distances and, when evaluating germination, we found a higher probability of germination after longer-distance pollination treatments. Therefore, the distance that pollen travels across the vereda area appears to somewhat influence the reproductive success of this species. We discuss these trends in detail below and explore their consequences for pollination biology and population ecology of the species in these threatened vereda plant formations.

Fruit and seed formation after short-distance hand pollinations highlight the absence of inbreeding barriers which has already been demonstrated for *C. spiralis* [42]. Fruit formation is possibly related to the abundance of *C. spiralis* flowers and natural pollination rates. Flower density affects pollen dispersal, as higher floral densities at the local scale are directly correlated with pollen-flow distance [43,44]. The somewhat clustered distribution of *C. spiralis* promotes an increased supply of resources for hummingbirds, which, in turn, tend to forage exclusively within these clumps [21,42,44]. The overwhelmingly high natural pollination rates at short distances [42] may have selected for lower inbreeding barriers, which could explain the fruit and seed set results.

At first glance, these results suggest that short-distance pollination is the main source of new seeds, as observed for other hummingbird-pollinated self-compatible plants [43,45]. In *Helicteris brevispira*, a hummingbird-pollinated self-compatible Cerrado shrub, most pollinator movements were less than 10 m and neighborhood areas (calculated from pollinators movements and seed dispersal estimates) were around 15 m in diameter [46]. When *H. brevispira* flower density was high in these clumps, hummingbirds were mostly territorial, thereby limiting pollen flow and creating genetic patches. Similarly, throughout our experiments, it was possible to observe the continuous visitation of *Eupetomena macroura* to *C. spiralis* flowers. *E. macroura* tends to have territorial behavior, staying near clumps with higher floral density, thus facilitating pollination within the clump. This hummingbird behavior was also observed in *Palicourea rigida*, another plant species from the Cerrado region [22,23].

Nevertheless, previous studies also reported the hummingbird *Phaethornis pretrei* as frequent pollinator of *C. spiralis* in the vereda [42], revealing a distinct behavior, in which pollinators visit the flowers of a group of individuals before moving to other groups further away. These hermit hummingbirds are less influenced by flower or resource density [24] and commonly show trapline foraging behavior [25]. This trapline foraging pattern can influence pollen deposition, extending pollen flow to other points along the vereda. This dynamic may help to explain why, despite heavier seeds at shorter distances, germination probability was higher after longer-distance hand pollinations. It suggests longer distances may provide seeds greater germination potential and agree with prior observations on increased seedling vigor [42]. Hermit hummingbird pollination may result in pollen flow between clumps along the vereda, which can lead to greater genetic variability, and somewhat explain the germination rate and seedling vigor [44,47,48]. Longer-distance pollinations commonly lead to greater genetic variability and heterozygote advantages, which have been described elsewhere [12,49,50,51]. Such differences could be further clarified using genetic markers to understand population structuring, as carried out elsewhere for other animal-pollinated Cerrado plants e.g., [15,46].

Ultimately, the pollen flow provided by different hummingbird species can be multimodal, involving dispersal over both short and long distances, reflecting the diverse strategies and behaviors of pollinators [17]. As mentioned earlier, the species that visit *C. spiralis* adopt different foraging behaviors, which can also be modified by environmental conditions or the availability of floral resources. In the case of *C. spiralis*, the plants are organized in clumps that vary in size and arrangement throughout most of the vereda. As a result, even territorial pollinators may occasionally forage between clumps of different sizes, leading to variable pollen-dispersal patterns [19]. Close relatives of *C. spiralis* were sampled in molecular analyses that detected low gene flow. The species *C. pulverulentus* and *C. scaber* are also pollinated by hummingbirds, which in theory have greater long-distance flight capabilities. However, *C. scaber* shows much greater isolation between populations in Costa Rica and Panama than *C. pulverulentus*, even though they have similar seed dispersal. This suggests that the difference could be explained by the behavior of their pollinators, which, in the case of *C. scaber*, are territorial [52]. 

Other Cerrado plants seem to use similar flexible, multimodal strategies. *Caryocar brasiliensis*, a bat-pollinated Cerrado tree, is self-compatible and adapted to inbreeding in disturbed areas, despite outbreeding being prevalent in the long term, shaping the natural population’s genetic structure [14]. Thus, outcrossing rates may vary widely in self-compatible species such as *C. spiralis* and may be affected by [differences in pollinator availability and floral display size [1,44,53]. The specificity of *Costus* pollinators contributes strongly to reproductive isolation among sympatric species studied across multiple habitats and years [54], often serving as a barrier to pollen flow between species. This broader Neotropical study also included *C. spiralis*, which was visited there by other hummingbird species, highlighting the importance of conducting studies in different habitats.

*Costus spiralis* in the vereda palm swamp is possibly restricted to pollen flow along these linear, moisture-rich areas, amid a much drier Cerrado savanna matrix. Hummingbirds connect *C. spiralis* clumps and facilitate pollen flow which may be vital for these plants in such relatively isolated habitats. Our findings demonstrate the complexity of the factors associated with the reproductive success of *C. spiralis*. We are aware that the population genetics of the species will help to clarify these trends, and such studies are already underway. The distribution of *C. spiralis* plants and the availability of floral resources may help elucidate pollen flow, and due to the flooded habitat, we are planning to use drone surveys as in [55] during the next flowering seasons. Finally, new techniques have been devised to mark pollen grains e.g., [53,56] which may allow direct measurement of pollen flow among *C. spiralis* clumps. Furthermore, it will be interesting in future studies to evaluate gene flow between different vereda areas in the region to identify the level of isolation of the studied populations caused by the territorial behavior of their most common pollinators.

Taken together, our findings indicate that the reproductive success of *C. spiralis* relies on a multimodal pollination system that balances short-distance efficiency with long-distance genetic exchange. This dual strategy may be essential for maintaining population resilience and genetic diversity in the fragmented and threatened vereda landscapes of the Cerrado.

## 4. Materials and Methods

### 4.1. Study Area

The present study was carried out in a preserved native area belonging to the Ecological Reserve of the Club “Caça e Pesca Itororó” (CCPIU). The CCPIU reserve is located in the municipality of Uberlândia, Minas Gerais state (48°18′06″ W and 18°59′21″ S). It covers approximately 400 ha of Cerrado, comprising various plant formations. The studied vereda area is protected as a Private Natural Reserve (RPPN). It is ca. 4 km long and encompasses around 100 ha [57]. The region is characterized by well-defined seasonality, with a cooler and drier season from May to September, and a warmer and rainy season from October to April. The climate of the region is classified as Aw Megathermic type [58].

### 4.2. Species Studied

The spiral ginger *Costus spiralis* is a rhizomatous herb that grows in clusters (clumps) of stems sprouting from underground rhizomes, which sometimes makes it difficult to distinguish individual genets. Each clump produces several terminal racemose spike inflorescences (up to 20 per clump) surrounded by overlapping red bracts, which protect the fascicles of developing floral buds, flowers and fruits. Flower and fruit maturation occur in an ascending spiral, with each inflorescence opening one or two flowers per day. The flowers possess a red tubular petaloid structure surrounding the single fertile stamen and the style, which grows between the two thecae of the anther and positions the stigma slightly above the stamen (approach herkogamy). The stigma is bilobed and moist, with one of the lobes a little shorter than the other. The stigma has a dorsal appendage that apparently functions to reposition itself after pollinator visits. This species is self-compatible, and its approach herkogamy ensures that the stigma is the first structure contacted by visitors attempting to reach the petaloid tube [42]. *Costus spiralis* is pollinated by several species of hummingbirds. The production of extrafloral nectar on the bracts means that its inflorescences are constantly visited and patrolled by ants [59], which appear to deter insect visitors but not the agile hummingbirds. Flowering begins at the end of January and lasts until June, peaking in February and March. Fruit production begins in July, and dispersal starts in August.

### 4.3. Hand-Pollination Tests with Different Distances and Evaluation of Reproductive Success

To investigate the effect of cross-pollination distance on the reproductive success of *C. spiralis* we carried out four hand-pollination treatments, each treatment corresponding to a distance between pollen donor and pollen recipient. Clumps of plants were considered as individual plants and studied in three different subpopulations in the vereda (A, B and C, Figure 1). We used eight plant clumps in area A, seven plant clumps in area B and three plant clumps in area C. The distances between plants used for pollination treatments were: 10 m (pollination within a subpopulation area and between nearby clumps at least 10 m apart), 60 m (pollination within an area but between clumps at least 60 m but always less than 100 m away), 800 m (pollination between clumps in subpopulation areas A and B distant ca. 800 m) and 2000 m (pollinations between clumps in subpopulation areas A and C distant ca. 2000 m). Pollinations were carried out with a mixture of pollen from different inflorescences and individuals respecting the defined distances. Experimental pollinations were carried out both ways between areas A and B and A and C, but not between areas B and C due to logistical constraints.

We selected these distances based on the distribution of the species along the vereda (Figure 1) and used 120 flowers for each pollination distance treatment. Clumps were treated as individual plants, and we ensured all treatments were applied to each individual, maintaining a similar number of treatments per clump. We used freshly opened flowers that had been previously bagged with nylon mesh. Using tweezers, we removed the anthers (emasculation) from freshly opened flowers and stored them in properly labeled test tubes. The anthers were transported to another location along the vereda as quickly as possible (within ten minutes) while avoiding desiccation to maintain pollen viability. To ensure freshness, pollen was generally carried in one direction per day.

Before cross-pollinations with the transported anthers, we emasculated the recipient flowers. After completing hand pollinations, we bagged back the inflorescences until fruits developed, approximately three months later. We conducted the hand-pollination tests over two reproductive years (2021 and 2022), treated here as a random factor. This approach provided more consistent data, as flower and fruit set were very low in some individuals during the first year. We acknowledge this as a limitation of our study. Because potential differences between years and individuals may have happened, these variables were treated as random effects in the statistical analyses (individual nested within year).

We collected separately the fruits from each treatment and transported them to the laboratory. We quantified the number fruits and seeds per fruit for each plant clump and distance treatment. We weighed the seeds using an analytical balance (BEL-ENGINEERING model M, precision 0.0001 g, Monza-Milan, Italy). We germinated the seeds in a controlled environment with 12 h of light exposure at a temperature of 24 °C. Due to the focus on the pollination distance treatments, after quantifying fruits and seeds per clump, we inadvertently mixed seeds from all fruits of each pollination distance, losing information on the exact plant of origin. This limitation prevented us from statistically analyzing germination differences at the plant level. However, since a similar number of treatments was conducted per clump, we consider that this did not substantially affect the overall analyses.

We randomly selected 30 seeds from each treatment and placed them on filter paper in Gerbox plates (JPROLAB, São Paulo, Brazil) containing 25 mL of distilled water. We observed seeds daily and evaluated germination upon cotyledon emergence. The seed germination study was interrupted after 45 days, as no additional germination was observed beyond 32 days.

### 4.4. Statistical Analysis

We applied generalized linear mixed models (GLMMs) to assess the impact of distance category (fixed factor and predictor variable) on fruit formation, number of seeds per fruit and seed weight (mass). The years, with individuals nested within each year, were included in the model as random factors. For seed weight, we also included the number of seeds per fruit as a covariate. We applied generalized linear models (GLMs) to assess the impact of distance category (fixed predictor variable) on seed germination (response variable). As mentioned earlier, seeds from different individuals were mixed and randomly selected for each distance treatment; thus, individual effects could not be evaluated.

The models were fitted using the package *glmmTMB* v.1.1.4 [60]. Overdispersion evaluation and model validation were carried out using residuals simulation, (1000 iterations) in the package *DHARMa* v.0.4.5 [61]. For fruit set and seed germination probabilities, we used a binomial distribution with a logit link function. A negative binomial distribution with an inverse link function (package *MASS* v.7.3.58.1) was used to analyze the number of seeds per fruit, and a Gaussian distribution with an identity link function was applied for seed weight.

We assessed model fit by examining residual distribution simulated with *DHARMa* and tested model significance using likelihood-ratio chi-square tests (*car* package v.3.1.1). Whenever significant results were found, post hoc analyses were performed using Tukey-adjusted pairwise contrasts in the *emmeans* v.1.8.4.1 package. Model-adjusted (predicted) means and 95% confidence intervals were back-transformed using the *ggeffects* v.1.1.5 package [62]. Visualizations were generated using *ggplot2* v.3.4.1 [63]. The package introduces a light jitter of gray points around box plots for enhanced visualization. Although these points do not represent raw data, we retained them for improved graphical clarity, as commonly seen in published works. All analyses were conducted using R software version 4.2.2 (R Core Team Vienna, Autria) [64].

## 5. Conclusions

*Costus spiralis* is a self-compatible species pollinated by hummingbirds and predominantly found in flooded palm swamp veredas of central Brazil. Experimental hand pollinations at different distances indicated that the reproductive success of *C. spiralis* is influenced, to some extent, by the distance of pollen flow along the vereda. We found that shorter distances between pollen donors and recipients resulted in heavier seeds, whereas greater distances enhanced seed germination success, suggesting that genetic diversity and seedling vigor benefit from pollen flow over longer distances.

These findings indicate that *C. spiralis* is adapted to a multimodal pollen dispersal strategy, benefiting from both territorial and traplining hummingbirds to optimize reproductive success. Understanding these mechanisms is crucial for assessing how typical vereda species maintain genetic diversity and reproductive success in these threatened environments.

## Data Availability

All data is available in the article.

## Supplementary Materials

The following supporting information can be downloaded at: https://www.mdpi.com/article/10.3390/plants14213266/s1, Table S1: Specific geographic coordinates of *Costus spiralis* plants used for the pollination experiments in the three subpopulations within the studied vereda palm swamp area.
