# Supplementary material for: Distance Matters: Assessing the Influence of Spatial Separation on Reproductive Success of Costus spiralis (Costaceae) in a Vereda Palm Swamp
Source: Plants (Basel). 2025 Oct 26;14(21):3266. doi: 10.3390/plants14213266 (PMC12609515; doi:10.3390/plants14213266)
Supplement: Supplementary file 1 [file plants-14-03266-s001.zip › plants-3879534-supplementary.pdf]

Supplementary Material

Table S1: Specific geographic coordinates of *Costus spiralis* plants used for the pollination experiments in the three subpopulations within the studied vereda palm swamp area.

Subpopulation	Individual	Latitude	Longitude
A	1	S19° 00.161	W48° 18.785
A	2	S19° 00.166	W48° 18.786
A	3	S19° 00.184	W48° 18.788
A	4	S19° 00.187	W48° 18.789
A	5	S19° 00.194	W48° 18.782
A	6	S19° 00.192	W48° 18.768
A	7	S19° 00.193	W48° 18.764
A	8	S19° 00.194	W48° 18.758
B	17	S18° 59.940	W48° 18.387
B	18	S18° 59.933	W48° 18.381
B	19	S18° 59.933	W48° 18.375
B	21	S18° 59.935	W48° 18.372
B	26	S18° 59.920	W48° 18.378
B	28	S18° 59.919	W48° 18.377
B	29	S18° 59.899	W48° 18.374
C	30	S18° 59.069'	W48° 17.803'
C	32	S 18°59'04"	W48°17'48"
C	33	S 18°59'04"	W 48°17'47"
